# Chronic Conditions and Patient-Centered Outcomes After Transcatheter Aortic Valve Intervention

**DOI:** 10.1016/j.jacadv.2025.102231

**Published:** 2025-10-17

**Authors:** Benjamin S. Wessler, Amanda Stebbins, Mohamad Alkhouli, Dharam J. Kumbhani, Tsuyoshi Kaneko, Vinod Thourani, Wayne Batchelor, Tamara Vesel, Cynthia Marturano, Carey Kimmelstiel, Charles Resor, Andrew Weintraub, Cynthia Boyd, Andrzej Kosinski, Sreekanth Vemulapalli, James E. Udelson, David M. Kent

**Affiliations:** a Division of Cardiology, Cardiovascular Center at Tufts Medical Center, Boston, Massachusetts, USA; b Duke Clinical Research Institute, Durham, North Carolina, USA; c Mayo Clinic Alix School of Medicine, Rochester, Minnesota, USA; d Division of Cardiology, UT Southwestern Medical Center, Dallas, Texas, USA; e Department of Cardiothoracic Surgery, Washington University in St. Louis, Missouri, USA; f Department of Cardiovascular Surgery, Marcus Heart and Vascular Center, Piedmont Heart Institute, Atlanta, Georgia, USA; g Inova Heart and Vascular Institute, Falls Church, Virginia, USA; h Division of Palliative Care at Tufts Medical Center, Boston, Massachusetts, USA; i Department of Medicine, General Internal Medicine at Tufts Medical Center, Boston, Massachusetts, USA; j Division of Cardiology, Cardiovascular Center at Tufts Medical Center, Boston, Massachusetts, USA; k Division of Cardiology, Cardiovascular Center at Tufts Medical Center, Boston, Massachusetts, USA; l Division of Cardiology, Cardiovascular Center at Tufts Medical Center, Boston, Massachusetts, USA; m Department of Medicine, Johns Hopkins University, Baltimore, Maryland, USA; n Duke Clinical Research Institute, Durham, North Carolina, USA; o Division of Cardiology, Duke Clinical Research Institute, Durham, North Carolina, USA; p Division of Cardiology, Cardiovascular Center at Tufts Medical Center, Boston, Massachusetts, USA; q Predictive Analytics and Comparative Effectiveness (PACE) Center, Tufts Medical Center Boston, Massachusetts, USA

**Keywords:** aortic stenosis, chronic conditions, older adults, transcatheter aortic valve intervention

## Abstract

**Background:**

Transcatheter aortic valve intervention (TAVI) has revolutionized the care of older adults with aortic stenosis.

**Objectives:**

The objectives of the study were to examine associations between chronic conditions and outcomes after TAVI and to describe palliative care utilization rates.

**Methods:**

This cohort study used the Society of Thoracic Surgeons/American College of Cardiology Transcatheter Valve Therapy registry to identify patients who underwent TAVI and were eligible for linkage with Centers for Medicare & Medicaid Services claims data. The exposure was multiple chronic conditions (MCCs) in the year before TAVI. Associations between chronic conditions and outcomes were assessed using multivariable logistic regression.

**Results:**

A total of 188,629 TAVI procedures were linked to Centers for Medicare & Medicaid Services claims. The median (IQR) age was 82.0 (76.0-87.0) years; 86,841 (46%) were female. Chronic conditions were associated with worse 1-year mortality (high MCC [≥6 conditions] vs low MCC [<4 chronic conditions], adjusted HR: 2.33 [95% CI: 2.22-2.44]). Chronic conditions were associated with lower Kansas City Cardiomyopathy Questionnaire at baseline (high MCC median score 37.5 [21.4-56.8] vs low MCC median score 55.7 [37.5-75.0], *P* < 0.001); however, the average improvement in Kansas City Cardiomyopathy Questionnaire after TAVI was large and appeared independent of chronic disease burden (median score change high MCC 28.7 [9.9-48.4] vs low MCC 24.5 [8.3-42.2], standardized difference +13.8%). Palliative care encounters were rare (8,946, 4.7%) and varied significantly across centers (range 0% to 25% of cases).

**Conclusions:**

Chronic conditions are associated with worse survival after TAVI. However, most patients with high MCC are alive 1 year after treatment, and quality of life improvements appear independent of chronic disease burden. These data help clarify expected health gains for patients with chronic conditions and symptomatic aortic stenosis.

Calcific aortic stenosis (AS) is a progressive form of valvular heart disease (VHD) that becomes increasingly prevalent with advancing age. Until the development of transcatheter aortic valve intervention (TAVI), there were limited treatment options for patients with prohibitive surgical risk. Today, TAVI enables treatment of patients with symptomatic AS and high procedural risk.

Current guidelines recommend aortic valve intervention for patients with symptomatic AS and life expectancy after TAVI ≥ 12 months.^1^ However, predicting individual life expectancy is challenging^2^^,^^3^ and defining futility remains difficult.^4^^,^^5^ Outcomes for high-risk patients treated with TAVI are particularly difficult to predict,^6^ underscoring the need for improved patient selection.^7^

Multiple chronic conditions (MCCs) are common for older adults^8^ although the burden of chronic conditions among patients treated with TAVI are unknown. Chronic conditions are known to negatively impact quality of life (QoL), functional status, and mortality^9^, ^10^, ^11^ and may be associated with decreased effectiveness of treatments.^12^ As TAVI is offered to more and sicker patients, there is an open question about whether some patients might have limited opportunity to benefit from TAVI since survival and QoL are limited by their high burden of chronic disease.

As interventions for older adults with VHD increase in scope and availability, TAVI provides an opportunity to study procedural decision-making and outcomes for the highest-risk older adults with VHD.^13^ The aim of this analysis was to explore how chronic disease burden associates with postprocedure QoL and mortality outcomes following TAVI.

## Methods

Data were collected from the Society for Thoracic Surgeons/American College of Cardiology Transcatheter Valve Therapy (STS/ACC TVT) registry for patients with severe symptomatic AS treated with a TAVI procedure between January 1, 2013, and December 31, 2020. The STS/ACC TVT registry serves as the national continued access registry for all TAVI procedures in the United States.^14^ The American College of Cardiology Foundation Institutional Review Board reviewed and approved the protocol in accordance with 45 CFR 46.116(d) of federal regulations. The Institutional Review Board waived the requirement for obtaining consent for the STS/ACC TVT registry. This study was approved by the Duke University Institutional Review Board and patient consent was waived because no new data were collected. Data collected by the STS/ACC TVT registry include patient demographic and clinical information, echocardiographic and laboratory data, procedural details, and patient outcomes.

### MCCs in patients undergoing TAVI

Claims data from hospital discharges and ambulatory physician services were used to identify the comorbid conditions contained in the Charlson-Deyo Score, Table 1.^15^ These 19 conditions were chosen because they have been widely studied across a number of clinical fields. The number and distribution of comorbidities contained in this score, present in the 1 year leading up to TAVI, were identified.

**Table 1:** Comorbid Conditions in the Year Before TAVI

Hypertension	168,014 (89.1)
CHF	146,480 (77.7)
Cardiac arrhythmias	133,067 (70.5)
Chronic pulmonary disease	88,090 (46.7)
Renal failure	75,836 (40.2)
Fluid and electrolyte disorders	60,687 (32.2)
Pulmonary vascular disease	56,308 (29.9)
Pulmonary circulation disorder	48,155 (25.5)
Complicated diabetes	43,200 (22.9)
Coagulopathy	31,580 (16.7)
Tumor	23,167 (12.3)
Psychosis	20,732 (11.0)
Dementia	12,746 (6.8)
Weight loss	11,164 (5.9)
Liver disease	7,068 (3.7)
Alcohol abuse	3,492 (1.9)
Hemiplegia	3,240 (1.7)
Metastatic cancer	1958 (1.0)
HIV/AIDS	80 (0.0)

Values are n (%). Comorbid conditions present in the year before TAVI. Claims data from hospital discharges and ambulatory physician services are used to identify the comorbid conditions contained in the Charlson-Deyo score.^15^

CHF = chronic heart failure; TAVI = transcatheter aortic valve intervention.

### High-risk definition

Procedural risk was assigned by the treating center based on the local Heart Team assessment. The “high-risk” definition for this study used the local Heart Team reason for procedure labels “high risk” and “extreme-risk’ on the STS/ACC TVT registry case form.

### Palliative care utilization

The data linkage with Centers for Medicare & Medicaid Services administrative claims identified palliative care (PC) claims encounters (International Classification of Diseases-9-Clinical Modification V66.7) for patients within 1 year of treatment with TAVI (either before or after treatment).

### Outcomes

The primary outcome was mortality at 30 days and 1 year. Secondary outcomes include disease specific QoL and 1-year home time following TAVI. These outcomes were selected because there is a growing body of literature showing that older adults prioritize short-term health gains over long-term survival when facing treatment decisions.^16^ The Kansas City Cardiomyopathy Questionnaire (KCCQ) is a patient-reported, disease-specific health status survey developed to measure symptoms and QoL in patients with heart failure and has been validated for patients with AS.^16^, ^17^, ^18^ The shortened 12-item version of the KCCQ was collected as part of the registry at baseline and 30 days after TAVI. The KCCQ assesses 4 domains related to VHD: physical limitation, symptom frequency, QoL, and social limitation and ranges from 0 to 100, with higher scores indicating less symptom burden and better QoL. Linguistically and culturally validated translations of the KCCQ were provided to non-English speakers during treatment. We treated the KCCQ score as a continuous variable, although scores are often categorized as very poor (<25), poor (25-49), fair (50-74), and good (≥75) QoL.^18^^,^^19^ Changes in the KCCQ of 5, 10, and 20 points correspond to small, moderate, or large improvements, respectively.^20^

The secondary outcome of days alive and out of the hospital after TAVI was also assessed.^21^

### Statistics and data analysis

We first described chronic conditions present in the year before TAVI. MCC burden was divided into tertiles based on the overall distribution of conditions to establish “low,” “medium,” and “high” MCC categories. Baseline clinical variables, STS predicted risk of mortality and in-hospital TVT–predicted mortality were described across tertiles of MCC. Continuous variables were presented as median and IQR or 25th and 75th percentiles. Categorical variables were presented as percentages and counts. Standardized differences with the low MCC category as reference are reported for specific analyses.

The associations between MCC and 30-day and 1-year mortality were studied using unadjusted and adjusted Cox regression for all patients and for “high-risk” patients. The assumption of proportional hazards was not met for 1-year mortality (Supplemental Table 1, Supplemental Figures 1, and 2). There was slight deviation for events occurring soon after the procedure and then later after TAVI between the MCC ≥6 group and the MCC <4 group. The clinical relevance of this violation was assessed as minimal. The presented results report the average hazard over 1 year after the TAVI procedure.

Unadjusted and adjusted models were created. The complete list of variables used in the fully adjusted models is shown in the Supplemental Appendix. HRs and 95% CIs were reported with the low MCC category as the reference group. Clustering of patients within sites was taken into account using the robust sandwich variance estimation method. The assumption of linearity was tested; in cases where it was violated, linear spline transformations were performed.

The unadjusted and adjusted associations of the MCC tertiles with change in KCCQ at 30 days were assessed using linear regression models among patients with baseline KCCQ data. KCCQ change scores were also calculated across MCC strata. To address missingness, patients with 30-day KCCQ data were weighted by the inverse of their probability of having KCCQ data to calculate unadjusted and adjusted least square mean KCCQ values across MCC categories. Multivariate linear regression included all adjustment measures included in the mortality models and also baseline KCCQ values.

One-year home time (median number of days alive and home) was assessed according to the MCC tertiles. The unadjusted and adjusted associations of MCC tertiles with 1-year home time were assessed using Poisson regression models with the log link function and the logarithm of length of post-TAVI follow-up as offset. The robust sandwich variance estimator was used to account for clustering of patients within sites and possible overdispersion. Incidence rate ratios (IRRs) with the 95% CIs using the low MCC group as the reference were reported. The adjusted models included the same covariates listed for the mortality models.

Given the large sample size, we report standardized differences between groups. The standardized difference is the difference in the mean of a variable between 2 groups divided by an estimate of the SD of that variable. For standardized differences, a value >10% is generally considered significant.^22^

## Results

**Figure 1:**
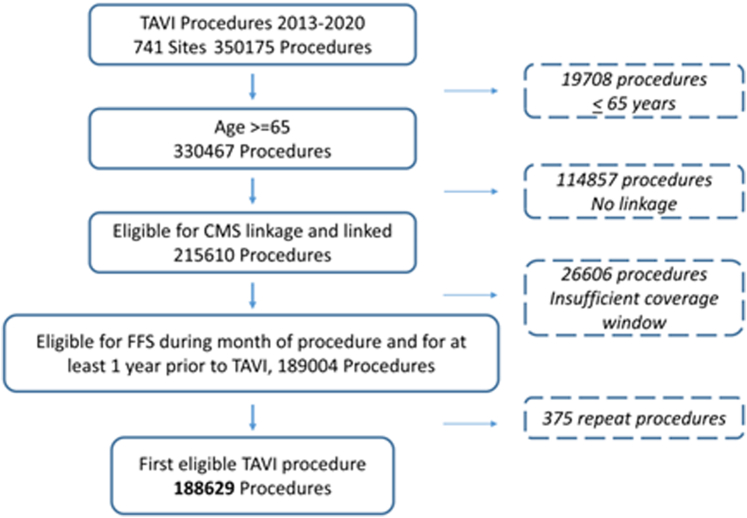
**Flowchart of Analytic Cohort** All TAVI procedures performed from 2013 to 2020 for patient ≥65 years old identified in the Society of Thoracic Surgeons/American College of Cardiology Transcatheter Valve Therapy (STS/ACC TVT) registry. Cases were enrolled in fee-for-service Medicare coverage during the procedural month and for at least 1 year before TAVI. Only first-ever TAVI was considered. CMS = Centers for Medicare & Medicaid Services; FFS = fee for service; TAVI = transcatheter aortic valve intervention.

A total of 350,175 TAVI procedures were performed across 741 sites from 2013 to 2020 (Central Illustration, Figure 1). Across 730 sites, 215,610 (61.6%) procedures were eligible for linkage to Centers for Medicare & Medicaid Services claims. Of these procedures, 189,004 (87.7%) were eligible for fee for service coverage during the month transcatheter aortic valve replacement took place and for at least 1 year before TAVI and were able to be linked to comorbidity data. A total of 188,629 (99.8%) first-time transcatheter aortic valve replacement procedures define the primary analytic cohort. The treating team considered 113,707 (60.3%) procedures as “high risk”. Baseline characteristics are shown in Table 2. The median age of patients was 82.0 (76.0-87.0) years; 46% were female. The median STS predicted risk of mortality was 5.2% (3.3%-8.3%). For both 30-day and 1-year mortality outcomes, we have complete follow-up. All available data are used for analysis and censored thereafter.

**Central Illustration:**
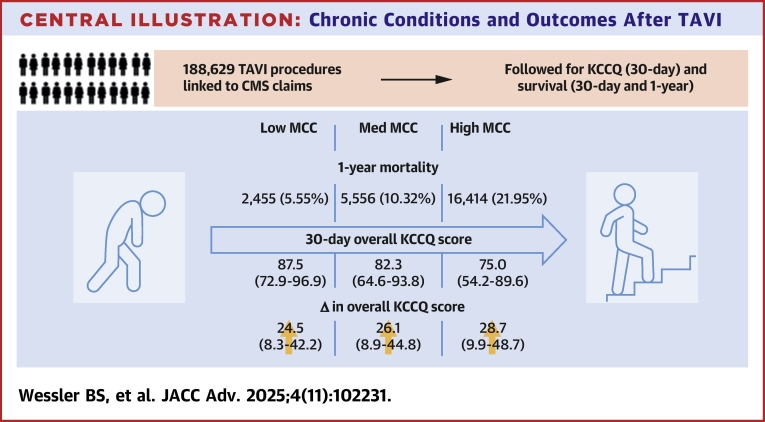
**Chronic Conditions and Outcomes After TAVI** TAVI procedures from the Thoracic Surgeons/American College of Cardiology Transcatheter Valve Therapy STS/ACC TVT Registry were linked to CMS claims and followed for short- and intermediate-term outcomes. MCC is multiple chronic conditions. CMS = Centers for Medicare & Medicaid Services; KCCQ = Kansas City Cardiomyopathy Questionnaire; MCC = multiple chronic condition; TAVI = transcatheter aortic valve intervention.

**Table 2:** Baseline Characteristics

	Overall (N = 188,629)	MCC <4 (n = 49,752)	MCC 4-<6 (n = 58,824)	MCC ≥6 (n = 80,053)
Age, median (IQR), y	82.0 (11)	82.0 (10)	82.0 (10)	82.0 (11)
Male (%)	101,788 (54)	25,912 (52.1)	31,735 (53.9)	44,141 (55.1)
Race (White), %	178,704 (95.7)	47,702 (96.8)	56,094 (96.2)	74,908 (94.6)
STS PROM (IQR), %	5.2 (5)	3.6 (3)	4.9 (4.2)	7.0 (6.5)
TVT PROM (IQR), %	3.2 (2)	2.6 (1.2)	3.1 (1.6)	3.8 (2.8)
Clinical variables number (%)				
LVEF (%)	60.0 (15)	60.0 (10)	60.0 (12)	57.0 (18)
eGFR mg/dL (IQR)	61.4 (33)	70.9 (27.4)	63.3 (31.1)	52.7 (33.2)
Dialysis	6,754 (3.6)	234 (0.5)	1,098 (1.9)	5,422 (6.8)
Prior MI	38,838 (20.6)	6,766 (13.6)	10,975 (18.7)	21,097 (26.4)
Prior stroke/TIA	32,901 (17.4)	6,709 (13.5)	9,946 (16.9)	16,246 (20.3)
PAD	50,113 (26.6)	8,267 (16.6)	14,658 (24.9)	27,188 (34.0)
Smoking	9,222 (4.9)	1882 (3.8)	2,676 (4.5)	4,664 (5.8)
Afib/flutter	73,773 (39.1)	11,360 (22.8)	21,914 (37.3)	40,499 (50.6)
Severe lung disease	17,186 (9.2)	1,698 (3.4)	4,426 (7.6)	11,062 (13.9)
Prior CABG	38,907 (20.6)	8,727 (17.5)	11,827 (20.1)	18,353 (22.9)
Nonfemoral access	15,884 (8.4)	1915 (3.8)	4,512 (7.7)	9,457 (11.8)
Acuity status number (%)				
Elective	169,259 (89.7)	47,602 (95.7)	54,208 (92.2)	67,449 (84.3)
Urgent	13,977 (7.4)	1,382 (2.8)	3,275 (5.6)	9,320 (11.6)
Preprocedure shock	4,570 (2.4)	663 (1.3)	1,139 (1.9)	2,768 (3.5)
Emergent/salvage	823 (0.4)	105 (0.2)	202 (0.3)	516 (0.6)

Characteristics according to chronic disease burden.

CABG = coronary artery bypass graft; eGFR = estimated glomerular filtration rate based on the CKD-EPI Creatinine Equation; LVEF = left ventricular ejection fraction; MCC = multiple chronic conditions; MI = myocardial infarction; PAD = peripheral artery disease; STS PROM = Society of Thoracic Surgeons predicted risk of mortality (30-day); TIA = transient ischemic attack; TVT PROM = transcatheter valve therapy predicted risk of mortality (in-hospital).

### Chronic conditions

The chronic conditions present in the year before TAVI are shown in Table 1. The median number of chronic conditions in the year before TAVI was 5 (IQR 3-7). Patients were divided into MCC tertiles (low MCC, <4 conditions, n = 49,752; medium MCC, 4 to <6 conditions, n = 58,824; high MCC, ≥6 conditions, n = 80,053). Of the patients, 25% had ≥7 chronic conditions.

The most common cardiovascular conditions were heart failure (146,480, 77.7%), hypertension (168,014, 89.1%), arrhythmias (133,067, 70.5%), chronic pulmonary disease (88,090, 46.7%), and renal failure (75,836, 40.2%). Less common conditions included tumors (23,167, 12.3%), psychosis (20,732, 11.0%), dementia (12,746, 6.8%), weight loss (11,164, 5.9%), liver disease (7,068, 3.7%), alcohol abuse (3,492, 1.9%), and metastatic cancer (1,958, 1.0%).

### MCC and mortality, overall cohort

**Figure 2:**
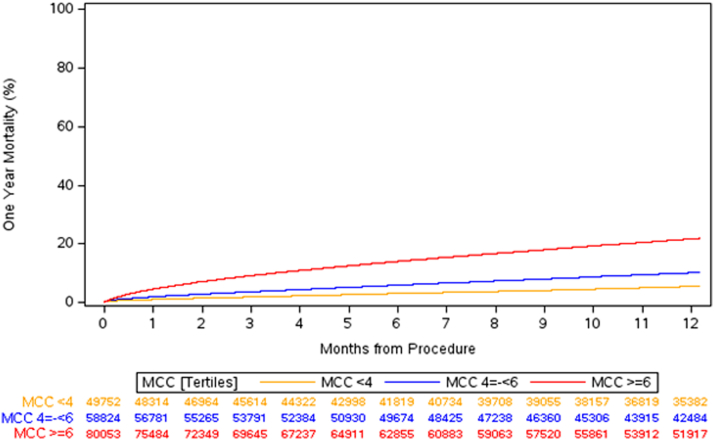
**1-Year Survival Curves for Transcatheter Aortic Valve Intervention Patients** Chronic disease burden is divided into tertiles. Low MCC (<4 conditions), intermediate MCC (4 to ≤6 conditions), and high MCC (>6 conditions). MCC = multiple chronic condition.

For all TAVI patients MCC was associated with worse 30-day and 1-year survival (Figure 2). 30-day mortality was 1.1% for low MCC vs 4.7% for high MCC (adjusted HR: 2.36; 95% CI: 2.13-2.60). One-year mortality was 5.6% for low MCC vs 22.0% for high MCC (adjusted HR: 2.33; 95% CI: 2.22-2.44) Table 3.

**Table 3:** Multiple Chronic Conditions and Mortality TAVI Patients: All-Cause Mortality Assessed at 30 and 365 Days for MCC-TAVI Patients

Follow Up	MCC <4	MCC 4-<6	MCC ≥6	Unadjusted Cox Model				Adjusted^a^ Cox Model			
	Event (CI Rate %)	Event (CI Rate %)	Event (CI Rate %)	Medium vs Low HR (95% CI)	*P* Value	High vs Low HR (95% CI)	*P* Value	Medium vs Low HR (95% CI)	*P* Value	High vs Low HR (95% CI)	*P* Value
30 d	543 (1.10%)	1,168 (1.99%)	3,706 (4.65%)	1.83 (1.65-2.02)	<0.001	4.30 (3.93-4.70)	<0.001	1.39 (1.26-1.55)	<0.001	2.36 (2.13-2.60)	<0.001
365 d	2,455 (5.55%)	5,556 (10.32%)	16,414 (21.95%)	1.91 (1.82-2.01)	<0.001	4.39 (4.21-4.58)	<0.001	1.45 (1.38-1.52)	<0.001	2.33 (2.22-2.44)	<0.001

Chronic conditions and 30-day and 1 year mortality. Table 3 represents the entire cohort Table 5 represents the “high-risk”.

MCC is multiple chronic conditions, stratified into tertiles. Comprehensive adjustment covariates are shown in the Supplemental Appendix.

TAVI = transcatheter aortic valve intervention; other abbreviations as in Table 1 and 2.

a Patients.

### MCC and KCCQ, overall cohort

**Figure 3:**
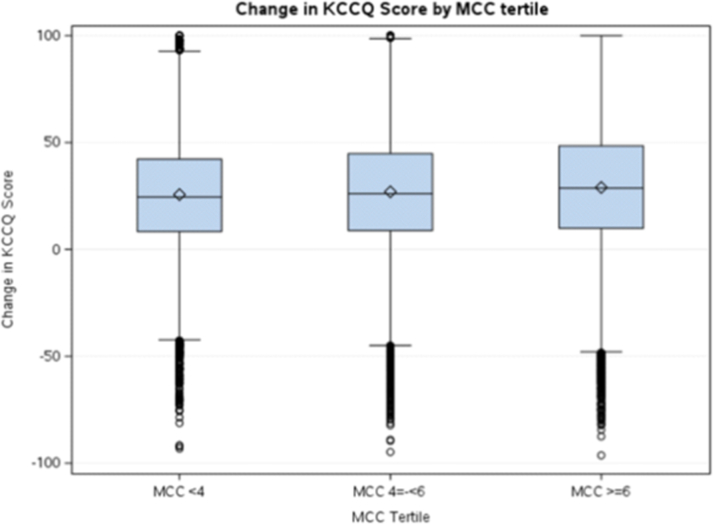
**Change in Quality of Life Stratified by Chronic Disease Burden** Multiple Chronic Conditions (MCC) divided into low MCC (<4 conditions), intermediate MCC (4 to <6 conditions), and high MCC (≥6 conditions). KCCQ is Kansas City Cardiomyopathy Questionnaire. The central horizontal line represents the median value. The box represents the interquartile range (IQR). Outlier values are represented by open circles. The central diamond represents the sample mean. KCCQ = Kansas City Cardiomyopathy Questionnaire.

For all TAVI patients MCC was associated with lower baseline KCCQ scores before TAVI (low MCC median 55.7 [37.5-75.0] vs high MCC median score 37.5 [21.4-56.8], standardized difference 65.2%). At 30 days, KCCQ data were available for 130,398 (71.0%) patients. Although patients with high MCC had lower 30-day KCCQ scores (75.0 [54.2-89.6] vs 87.5 [72.9-96.9], standardized difference 56.2%), similar large improvements in KCCQ were seen across all tertiles of MCC (high MCC median score change 28.7 [9.9-48.4] vs low MCC 24.5 [8.3-42.2], standardized difference 13.8%) (Figure 3, Table 4). To address KCCQ missingness, patients with 30-day KCCQ data were weighted by the inverse of their probability of having KCCQ data. Unadjusted and adjusted least square mean KCCQ values across MCC categories showed similar large improvement trends (Supplemental Appendix).

**Table 4:** KCCQ Stratified by Burden of Chronic Conditions for TAVI Patients: KCCQ Assessed at Baseline and 30 Days for TAVI Patients

					Standard Difference	
Follow Up	Overall (N = 173,492)	MCC <4 (n = 49,752)	MCC 4-<6 (n = 54,594)	MCC ≥6 (n = 72,422)	MCC 4-<6	MCC ≥6
Baseline overall KCCQ score						
Median (IQR)	45.8 (27.6-65.5)	55.7 (37.5-75.0)	47.9 (30.2-67.4)	37.5 (21.4-56.8)	27.7	65.2
Missing (%)	8.0	6.6	7.2	9.5		
30 d overall KCCQ score						
Median (IQR)	81.3 (62.5-93.8)	87.5 (72.9-96.9)	82.3 (64.6-93.8)	75.0 (54.2-89.6)	26.91	56.20
Missing (%)	25.2	22.4	23.6	28.3		
Change in KCCQ score						
Median (IQR)	26.6 (9.0-45.8)	24.5 (8.3-42.2)	26.1 (8.9-44.8)	28.7 (9.9-48.7)	5.96	13.81
Missing (%)	29.0	25.7	27.1	32.5		

KCCQ scores across MCC strata for TAVI patients (overall cohort). N is the total number of KCCQ assessments for each cohort. Values are shown as median (IQR). Missingness is described as a % of total TAVI performed in each stratum. Standard differences are shown with MCC *<*4 as the reference class. The standardized difference is not influenced by sample size, unlike the *P* value. Standardized differences ranging between −10 and 10 are thought insignificant.

KCCQ = Kansas City Cardiomyopathy Questionnaire; other abbreviations as in Table 1 and 2.

### MCC and mortality, high-risk cohort

For the “high-risk” TAVI patients similar trends were seen. 30-day mortality was 1.6% for low MCC vs 5.5% for high MCC (adjusted HR: 2.10; 95% CI: 1.87-2.37). For “high-risk” TAVI patients 1-year mortality was 7.7% for low MCC vs 24.6% for high MCC (adjusted HR: 2.08; 95% CI: 1.96-2.20) (Supplemental Figure 3, Table 5).

**Table 5:** Multiple Chronic Conditions and Mortality TAVI Patients: All-Cause Mortality Assessed at 30 and 365 Days for MCC-TAVI Patients: High-Risk Patients

Follow Up				Unadjusted Cox Model				Adjusted Cox Model^a^			
	MCC < 4 Event (CI Rate %)	MCC 4-<6 Event (CI Rate %)	MCC ≥6 Event (CI Rate %)	Medium vs Low HR (95% CI)	*P* Value	High vs Low HR (95% CI)	*P* Value	Medium vs Low HR (95% CI)	*P* Value	High vs Low HR (95% CI)	*P* Value
30 d	361 (1.63%)	884 (2.63%)	3,163 (5.49%)	1.62 (1.43-1.83)	<0.001	3.42 (3.06-3.81)	<0.001	1.29 (1.14-1.46)	<0.001	2.10 (1.87-2.37)	<0.001
365 d	1,625 (7.74%)	4,108 (12.82%)	13,548 (24.62%)	1.70 (1.61-1.80)	<0.001	3.53 (3.35-3.72)	<0.001	1.35 (1.28-1.43)	<0.001	2.08 (1.96-2.20)	<0.001

Chronic conditions and 30-day and 1 year mortality. Table 3 represents the entire cohort Table 5 represents the “high-risk”.

MCC is multiple chronic conditions, stratified into tertiles. Comprehensive adjustment covariates are shown in the Supplemental Appendix.

TAVI = transcatheter aortic valve intervention; other abbreviations as in Table 1 and 2.

a Patients.

### MCC and KCCQ, high risk cohort

Similar KCCQ patterns were seen for “high-risk” TAVI patients. For “high risk” patients, MCC was associated with lower baseline KCCQ scores before TAVI (low MCC median 51.0 [33.3-69.8] vs high MCC median score 35.4 [19.8-54.2], standardized difference 57.7%) (Table 6). At 30 days, KCCQ data were available for 75,393 (68.8%) patients. The median 30-day KCCQ score was lower for high MCC patients compared to low MCC patients (72.9 [52.1-88.2] vs 84.0 [67.7-94.8], standardized difference 46.4%). Large average improvements in KCCQ were seen across all tertiles of MCC (high MCC median score change 29.2 [9.7-49.0] vs low MCC 25.5 [8.9-43.8], standardized difference 10.9%). 30-day unadjusted and adjusted weighted mean KCCQ scores showed similar trends (Supplemental Appendix).

**Table 6:** KCCQ Stratified by Burden of Chronic Conditions for TAVI Patients: KCCQ Assessed at Baseline and 30 Days for ‘High-Risk’ TAVI Patients

Outcome	N = 103,196	N = 20,515	N = 30,936	N = 51,745	MCC 4-<6	MCC ≥6
Baseline overall KCCQ score						
Median (IQR)	41.2 (24.0-60.4)	51.0 (33.3-69.8)	44.3 (27.1-63.5)	35.4 (19.8-54.2)	23.69	57.66
Missing (%)	9.2	7.6	8.3	10.4		
30 d overall KCCQ score						
Median (IQR)	77.1 (57.3-91.7)	84.0 (67.7-94.8)	79.2 (60.4-91.7)	72.9 (52.1-88.2)	21.48	46.37
Missing (%)	27.0	24.2	25.2	29.3		
Change in KCCQ score						
Median (IQR)	27.6 (9.4-46.9)	25.5 (8.9-43.8)	27.1 (8.9-45.8)	29.2 (9.7-49.0)	3.93	10.94
Missing (%)	31.2	27.8	29.1	33.8		

KCCQ scores across MCC strata for “high-risk” TAVI patients. N is the total number of KCCQ assessments for each cohort. Values are shown as median (IQR). Missingness is described as a % of total TAVI performed in each stratum. Standard differences are shown with MCC <4 as the reference class. The standardized difference is not influenced by sample size, unlike the *P* value. Standardized differences ranging between −10 and 10 are thought insignificant.

KCCQ = Kansas City Cardiomyopathy Questionnaire; other abbreviations as in Table 1 and 2.

### MCC and days alive and out of the hospital

Patients with high MCC experienced fewer days alive out of the hospital in the year after TAVI compared to patients with low MCC. For the overall cohort, the median number of days alive out of hospital 340.0 vs 360.0, IRR: 0.94 (0.95, 0.94); *P* < 0.001. For the “high risk” cohort, the median number of days alive out of hospital 348.0 vs 360.0, IRR: 0.94 (0.94, 0.94); *P* < 0.001 (Table 7).

**Table 7:** Days Alive and Out of the Hospital for High-Risk TAVI Patients

	High Risk Cohort: Days Alive and out of the Hospital for MCC-TAVI Patients						
	Median Number of Days Alive and Out of Hospital	Unadjusted			Adjusted Model^b^		
		Incidence Rate Ratio (95% CI)^a^	Z Statistic	*P* Value	Incidence Rate Ratio (95% CI)^a^	Z Statistic	*P* Value
MCC <4	360.0						
MCC 4-<6 vs MCC <4	358.0	0.98 (0.98-0.98)	−64.8	<0.0001	0.99 (0.99-0.99)	−14.9	<0.0001
MCC ≥6 vs MCC <4	348.0	0.94 (0.94-0.94)	−26.9	<0.0001	0.96 (0.96-0.96)	−41.0	<0.0001

Abbreviations as in Table 1 and 2.

a Reference group is MCC <4.

b Adjustment covariates are shown in the Supplemental Appendix.

### Palliative care claims

The percentage of TAVI procedures with a PC claims varied widely across 729 sites. The median PC use rate was 3.5% (IQR: 2.9%) although the PC use ranged from 0% to 25% of TAVI cases across centers. PC use was associated with chronic disease burden (1.8% for low MCC vs 7.6% for high MCC). Overall, PC use was associated with 87% mortality at 1 year.

## Discussion

The primary findings from this analysis are that chronic conditions are common for patients treated with TAVI and are associated with worse outcomes after treatment, including higher mortality and lower QoL at 30 days. Nevertheless, the magnitude of improvements in QoL in the highest MCC group is large and appear similar to other patients. Most patients are alive 1 year after TAVI, including those classified as “high-risk” TAVI patients with high MCC. These results support the potential for clinical benefit of TAVI even for the highest-risk patients with significant comorbid illness and emphasize the importance of eliciting patient-defined treatment goals.

Chronic conditions are increasingly recognized as an important consideration for older adults with CVD.^23^ Standard risk scores often do not account for important chronic conditions and although MCC negatively impacts QoL, functional status, and mortality,^9^, ^10^, ^11^ the impact on therapeutic benefit for specific treatments appears less certain.^24^ Trials of TAVI have largely excluded patients with significant competing risks (ie, end stage renal disease or expected survival <12 months), although real-world patients are often more complex than those enrolled in clinical trials.^25^ To our knowledge, there has not been a comprehensive description of chronic conditions for TAVI trial participants. Although comorbid illness may be a common reason for withholding TAVI,^26^ this real-world study demonstrates that most patients treated with TAVI (during the high-risk treatment era) have a substantial burden of chronic conditions. Although MCC associates with worse outcomes, MCC burden does not appear helpful in identifying patients unlikely to benefit from TAVI. To the contrary, on average, large improvements in symptom burden as measured by the KCCQ appear independent of chronic disease burden.

High-risk patients are commonly offered TAVI and potential benefit is often framed in terms of increased survival. Prolonged survival may not be the most important to many older adults who often prioritize symptom improvement over other short-term goals.^27^ This analysis suggests that most high-risk patients experience significant improvement in symptoms after TAVI, and that these patients—independent of chronic disease burden—might very well achieve their treatment goals. We also found that AS patients with metastatic disease or dementia are occasionally treated with TAVI, a finding that suggests this procedure might be offered primarily for symptom improvement (not mortality benefit) to certain patients. Although this may be an appropriate treatment for some, such an approach should only be considered in the context of comprehensive end-of-life care. These results do not offer insights about the cost-effectiveness of such an approach.

Although this study focuses on chronic disease burden, some of the most actionable observations for older adults relate to other geriatric assessments that are associated with short- and long-term outcomes and can help improve the patient-centeredness of treatment decisions.^28^ Frailty, defined as a measure of vulnerability to future stressors, can be quantified at the bedside and is associated with worse outcomes after TAVI.^29^ A comprehensive geriatric assessment represents a more thorough assessment of medical, physical, cognitive, social, and financial conditions that associate with worse outcomes for older adults. Although comprehensive geriatric assessment can help optimize treatment decisions for older adults, implementing these more comprehensive assessments remains a challenge. Formal considerations of the geriatric syndromes that associate with severe AS will help clinicians arrive at more patient-centered treatment decisions.

Palliative care is recommended early in the clinical course for patients with advanced CVD.^30^ Many high-risk patients with symptomatic AS (regardless of the decision for or against TAVI) experience high mortality, frequent persistent symptoms, and the stress of living with a serious illness. Although most providers hold favorable views of PC consultation in the setting of TAVI, it is rarely offered to those who are ineligible for TAVI.^31^ Our results are the first to demonstrate that PC encounter rates are low even for high-risk TAVI patients despite receiving TAVI near the end of life. Even in the setting of caring for “high-risk” symptomatic AS patients, PC encounters occur at rates that are well below those seen for advanced HF. Further research is needed to understand whether PC interventions either before or after high-risk TAVI improve patient-centered outcomes.

### Study Limitations

There are several limitations of this study. First, MCC is based on claims data and a simple count does not capture variation in comorbidity type and severity or duration. However, this approach allows for the development of simplified risk schemes into electronic medical records. Second, these results pertain to an earlier treatment era where TAVI was reserved for intermediate and high-risk patients. Third, there are no data on use of guideline-directed medical therapy following TAVI, particularly for patients with heart failure. Fourth, approximately 25% of patients are missing KCCQ data at 30 days. Fifth, primary PC delivered by cardiologists is likely missed in our assessment and plays an important role in addressing end-of-life care concerns. Finally, it is difficult to evaluate the degree to which patients benefit from the treatment in a single-arm observational study, with no controls. For example, we could not compare the benefit in mortality reduction that patients received from TAVI across levels of comorbidity burden.

Nevertheless, in terms of the absolute improvement in baseline symptoms, as captured by the KCCQ, high MCC patients appear to have a similar degree of symptomatic improvement as other patients with lower MCC burden at 30 days. Whether these improvements are sustained beyond this timeframe cannot be determined.

## Conclusions

Chronic conditions are common among patients treated with TAVI and are associated with worse outcomes. However, substantial symptomatic improvement is common and is seen across varying levels of chronic disease burden. The similar large change in KCCQ scores across MCC categories suggests that even high-risk patients with significant comorbid conditions may attain significant symptomatic benefit from TAVI. These data may inform shared decision-making discussions with patients.

Perspectives **COMPETENCY IN MEDICAL KNOWLEDGE 1:** This study provides intermediate-term outcome data up to 1 year for patients treated with TAVI. Findings show chronic disease burden is associated with worse outcomes after TAVI; however most patients are alive 1 year after treatment.

**COMPETENCY IN MEDICAL KNOWLEDGE 2:** Patients with significant comorbid disease at the time of TAVI have lower disease-specific QoL measures; however, average improvement in QoL appears significant and independent of comorbid disease burden.

**TRANSLATIONAL OUTLOOK:** Future research should focus on chronic disease burden associations with longer-term assessments of QoL after TAVI and how predictions align with treatment goals for older adults with AS.

## Funding support and author disclosures

Dr Wessler was supported by K23AG055667 (10.13039/100000002NIH/10.13039/100000049NIA) and UL1TR002544 (10.13039/100000002NIH/10.13039/100006108NCATS); and has done consulting work for iCardio.ai unrelated to the present work. Dr Kaneko is in the advisory board of Edwards, Abbott, 4C Medical, and Anteris and is a consultant at Medtronic. Dr Boyd coauthored a chapter on multiple chronic conditions for UpToDate. All other authors have reported that they have no relationships relevant to the contents of this paper to disclose.

## References

[1] Otto C.M., Nishimura R.A., Bonow R.O. (2021). 2020 ACC/AHA guideline for the management of patients with valvular heart disease: a report of the American College of Cardiology/American Heart Association joint committee on clinical practice guidelines. J Am Coll Cardiol.

[2] Christakis N.A., Lamont E.B. (2000). Extent and determinants of error in doctors’ prognoses in terminally ill patients: prospective cohort study. BMJ.

[3] Alba A.C., Buchan T.A., Saha S. (2024). Factors impacting physician prognostic accuracy in heart failure patients with reduced left ventricular ejection fraction. JACC Heart Fail.

[4] Lindman B.R., Alexander K.P., O’Gara P.T., Futility A.J. (2014). Benefit, and transcatheter aortic valve replacement. JACC Cardiovasc Interv.

[5] Wessler B.S., Weintraub A.R., Udelson J.E., Kent D.M. (2020). Can clinical predictive models identify patients who should not receive TAVR? A systematic review. Struct Heart J Hear Team.

[6] Arnold S.V., Spertus J.A., Vemulapalli S. (2017). Quality-of-Life outcomes after Transcatheter aortic valve replacement in an unselected population. JAMA Cardiol.

[7] Holmes D.R., Brennan J.M., Rumsfeld J.S. (2015). Clinical outcomes at 1 year following transcatheter aortic valve replacement. JAMA.

[8] Ritchie C.S., Zulman D.M. (2013). Research priorities in geriatric palliative care: multimorbidity. J Palliat Med.

[9] Wolff J.L., Starfield B., Anderson G. (2002). Prevalence, expenditures, and complications of multiple chronic conditions in the elderly. Arch Intern Med.

[10] Fortin M., Bravo G., Hudon C. (2006). Relationship between multimorbidity and healthrelated quality of life of patients in primary care. Qual Life Res.

[11] Lee T.A., Shields A.E., Vogeli C. (2007). Mortality rate in veterans with multiple chronic conditions. J Gen Intern Med.

[12] Weiss C.O., Varadhan R., Puhan M.A. (2014). Multimorbidity and evidence generation. J Gen Intern Med.

[13] Damluji A.A., Nanna M.G., Rymer J. (2024). Chronological vs biological Age in interventional cardiology: a comprehensive approach to care for older adults: JACC family series. JACC Cardiovasc Interv.

[14] Mack M.J., Brennan J.M., Brindis R. (2013). Outcomes following transcatheter aortic valve replacement in the United States. JAMA.

[15] Deyo R.A., Cherkin D.C., Ciol M.A. (1992). Adapting a clinical comorbidity index for use with ICD-9CM administrative databases. J Clin Epidemiol.

[16] Green C.P., Porter C.B., Bresnahan D.R., Spertus J.A. (2000). Development and evaluation of the Kansas City cardiomyopathy questionnaire: a new health status measure for heart failure. J Am Coll Cardiol.

[17] Pettersen K.I., Reikvam A., Rollag A., Stavem K. (2005). Reliability and validity of the Kansas City cardiomyopathy questionnaire in patients with previous myocardial infarction. Eur J Heart Fail.

[18] Arnold S.V., Spertus J.A., Lei Y. (2013). Use of the Kansas City cardiomyopathy questionnaire for monitoring health status in patients with aortic stenosis. Circ Heart Fail.

[19] Soto G.E., Jones P., Weintraub W.S., Krumholz H.M., Spertus J.A. (2004). Prognostic value of health status in patients with heart failure after acute myocardial infarction. Circulation.

[20] Spertus J., Peterson E., Conard M.W. (2005). Monitoring clinical changes in patients with heart failure: a comparison of methods. Am Heart J.

[21] Mentias A., Keshvani N., Desai M.Y. (2022). Risk-adjusted, 30-Day home time after Transcatheter aortic valve replacement as a hospital-level performance metric. J Am Coll Cardiol.

[22] Austin P.C. (2009). Using the standardized difference to compare the prevalence of a binary variable between two groups in observational research. Commun Stat - Simul Comput.

[23] Forman D.E., Maurer M.S., Boyd C. (2018). Multimorbidity in older adults with cardiovascular disease. J Am Coll Cardiol.

[24] Hanlon P., Butterly E.W., Shah A.S. (2023). Treatment effect modification due to comorbidity: individual participant data meta-analyses of 120 randomised controlled trials. PLoS Med.

[25] Buffel du Vaure C., Dechartres A., Battin C., Ravaud P., Boutron I. (2016). Exclusion of patients with concomitant chronic conditions in ongoing randomised controlled trials targeting 10 common chronic conditions and registered at ClinicalTrials.gov: a systematic review of registration details. BMJ Open.

[26] Tang L., Gössl M., Ahmed A. (2018). Contemporary reasons and clinical outcomes for patients with severe, symptomatic aortic stenosis not undergoing aortic valve replacement. Circ Cardiovasc Interv.

[27] Coylewright M., Palmer R., O’Neill E.S., Robb J.F., Fried T.R. (2016). Patient-defined goals for the treatment of severe aortic stenosis: a qualitative analysis. Heal Expect.

[28] Braun L.T., Grady K.L., Kutner J.S. (2016). Palliative care and cardiovascular disease and stroke: a policy statement from the American Heart Association/American Stroke Association. Circulation.

[29] Kirkpatrick J.N., Hauptman P.J., Swetz K.M. (2016). Palliative care for patients with EndStage cardiovascular disease and devices: a report from the palliative care working group of the geriatrics section of the American College of Cardiology. JAMA Intern Med.

[30] Mandawat A., Heidenreich P.A., Mandawat A., Bhatt D.L. (2016). Trends in palliative care use in veterans with severe heart failure using a large national cohort. JAMA Cardiol.

[31] Godfrey S., Kirkpatrick J.N., Kramer D.B., Sulistio M.S. (2023). Expanding the paradigm for cardiovascular palliative care. Circulation.

