# Supplementary material for: Chronic Conditions and Patient-Centered Outcomes After Transcatheter Aortic Valve Intervention
Source: JACC Adv. 2025 Oct 17;4(11):102231. doi: 10.1016/j.jacadv.2025.102231 (PMC12556185; doi:10.1016/j.jacadv.2025.102231)
Supplement: Supplemental_Material [file mmc1.pdf]

Supplement

All models were adjusted for the following variables: Age, sex, body surface area, left ventricular ejection fraction, baseline hemoglobin, baseline platelet count, procedure date, eGFRcr, current dialysis, non-white or Hispanic, left main stenosis >=50%, proximal LAD >=70%, prior MI, endocarditis, prior stroke or TIA, carotid stenosis, prior PAD, smoking status, diabetes, NYHA class IV, atrial fib/flutter, conduction defect, severe chronic lung disease, home oxygen use, hostile chest, carotid artery stenosis, porcelain aorta, pacemaker, previous ICD, prior PCI, prior CABG, prior cardiac operations, prior aortic valve procedure, prior mitral valve procedure, aortic etiology [degenerative vs. other], valve morphology [tricuspid vs other], moderate or severe aortic stenosis, moderate or severe mitral regurgitation, moderate or severe tricuspid regurgitation, non-femoral access and acuity of TAVI.

Supplemental Table 1:

Analysis of Maximum Likelihood Estimates					
Parameter	Parameter Estimate	Standard Error	Chi-Square	Pr > ChiSq	Hazard Ratio
MCC 4=-<6	0.64822	0.02423	715.4983	<.0001	1.912
MCC >=6	1.47932	0.02164	4673.6768	<.0001	4.390
zph Tests for Nonproportional Hazards					
	Correlation	ChiSquare	Pr > ChiSquare	t Value	Pr > |t|
MCC 4=-<6	-0.00152	0.0568	0.8117	-0.24	0.8117
MCC >=6	-0.0233	13.2275	0.0003	-3.64	0.0003

Supplemental Figure 1: Schoenfield Residuals

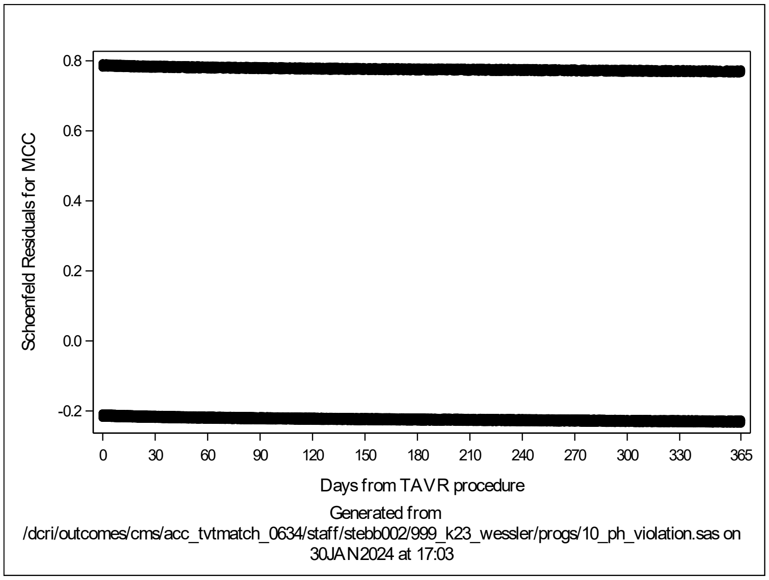

Supplemental Figure 2: Time-Varying Coefficient Plot MCC >=6 and one year mortality

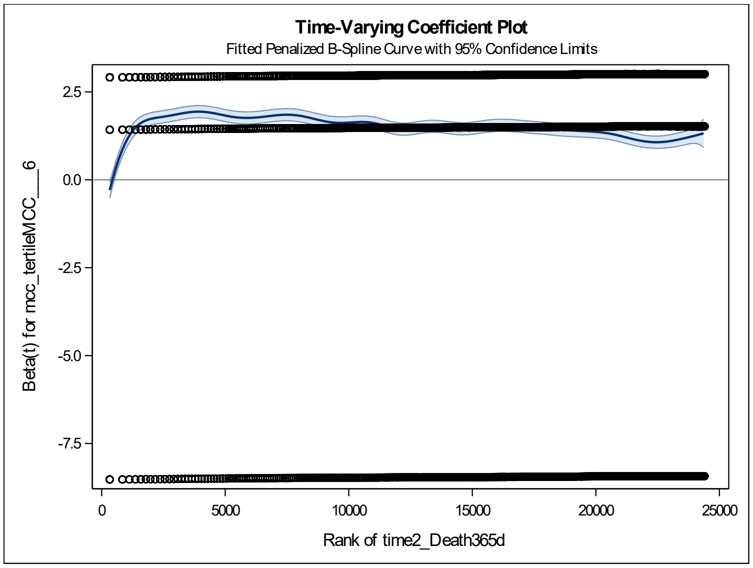

Supplement Figure 3:

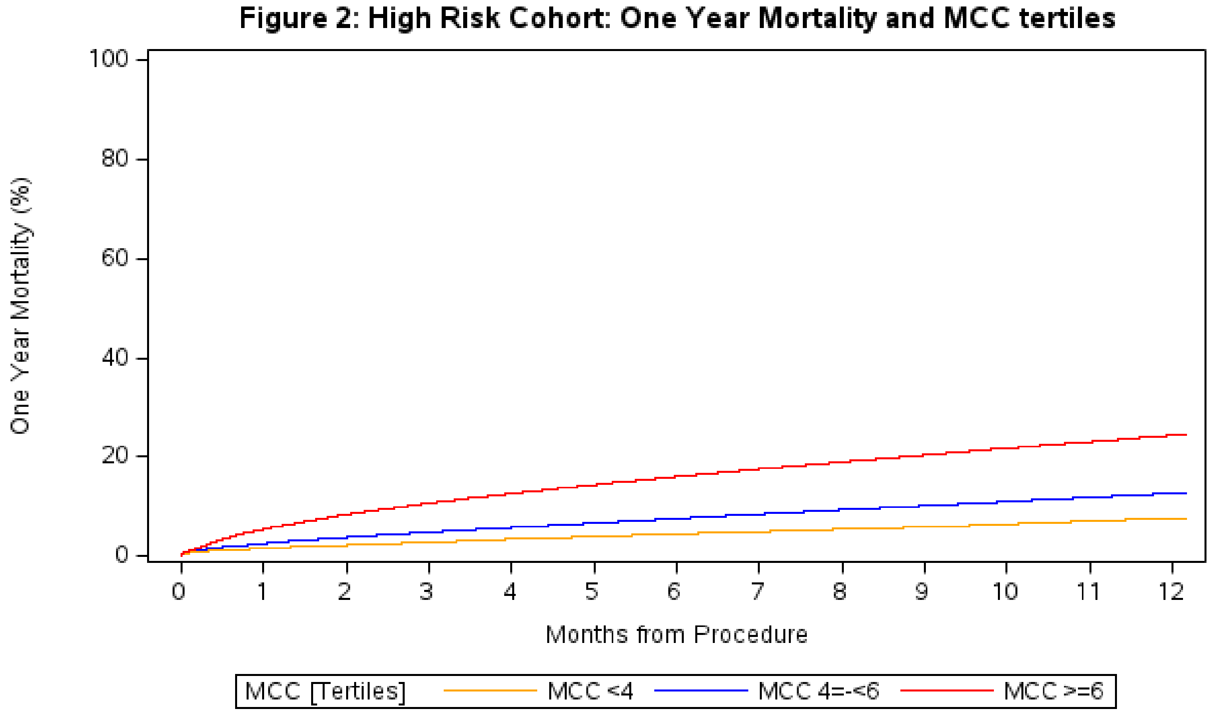

MCC <4	22194	21629	21236	20848	20464	20128	19798	19489	19189	18975	18690	18278	17933
MCC 4=-<6	33727	32553	31823	31152	30487	29847	29250	28658	28104	27674	27158	26561	25960
MCC >=6	57782	54168	51900	49991	48364	46808	45387	44060	42863	41808	40682	39419	38159

Generated from
/dcri/outcomes/cms/acc_tvtmatch_0634/staff/stebb002/999_k23_wessler/graphs/02_high_risk_cum_inc_1yr_dth.sas on 22JAN2024 at 09:39

Supplemental Table 2: KCCQ Scores by MCC tertiles (Full Cohort)

Variable	Level	Overall (N=188629)		MCC <4 (N=49752)		MCC 4-<6 (N=58824)		MCC >=6 (N=80053)		Std Dif Score* (MCC <4)	
										MCC 4-<6	MCC >=6
Baseline overall KCCQ score /ranks	Median	173492	45.8	46476	55.7	54594	47.9	72422	37.5	27.70	65.24
	25th		27.6		37.5		30.2		21.4		
	75th		65.6		75.0		67.4		56.8		
	Mean		47.3		55.9		49.2		40.3		
	STD		24.9		24.1		24.3		23.9		
	Missing(%)		8.0		6.6		7.2		9.5		
30 Day overall KCCQ score for eligible procedures /ranks	Median	137216	81.3	38187	87.5	44082	82.3	54947	75.0	26.91	56.20
	25th		62.5		72.9		64.6		54.2		
	75th		93.8		96.9		93.8		89.6		
	Mean		75.4		81.9		76.6		70.0		
	STD		22.4		19.0		21.4		24.0		
	Missing(%)		25.2		22.4		23.6		28.3		
Change in KCCQ score for eligible procedures /ranks	Median	130398	26.6	36602	24.5	42063	26.1	51733	28.7	5.96	13.81
	25th		9.0		8.3		8.9		9.9		
	75th		45.8		42.2		44.8		48.4		
	Mean		27.4		25.6		26.9		28.9		
	STD		26.2		24.3		25.9		27.6		
	Missing(%)		29.0		25.7		27.1		32.5		

Supplemental Table 3. KCCQ at 30 Day for MCC-TAVI patients (Full Cohort)

	Unadjusted	Adjusted	Unadjusted KCCQ			Adjusted[1] KCCQ Model		
	LS Mean (weighted)	LS Mean (weighted)	Estimate* (95% CI)	T Statistic	P Value	Estimate* (95% CI)	T Statistic	P Value
MCC <4	82.58	78.27						
MCC 4-<6	77.47	75.54	-5.11 (-5.40, -4.82)	-34.80	<.001	-2.73 (-3.02, -2.44)	-18.70	<.001
MCC >=6	70.93	71.97	-11.65 (-11.93, -11.36)	-80.00	<.001	-6.30 (-6.62, -5.97)	-38.20	<.001

*Reference group is MCC <4

[1] Adjusted by baseline KCCQ score, age, sex, BSA, lvef, hemoglobin, platelet count, procedure date, eGFRcr, current dialysis, non-white or Hispanic, left main stenosis >=50%, proximal LAD >=70%, Prior MI, endocarditis, prior stroke/tia, carotid stenosis, prior PAD, smoking status, diabetes, NYHA class IV, atrial fib/flutter, conduction defect, severe chronic lung disease, home oxygen use, hostile chest, porcelain aorta, pacemaker, previous ICD, prior PCI, prior CABG, prior cardiac operations, prior aortic valve procedure, prior mitral valve procedure, aortic etiology [degenerative vs. other], valve morphology [tricuspid vs other], moderate/severe aortic stenosis, moderate/severe mitral regurgitation, moderate/severe tricuspid regurgitation, non-femoral access and acuity of TAVR [elective, urgent, shock or inotropes or assist device, emergency or salvage or cardiac arrest].

Supplemental Table 4. KCCQ at 30 Days after TAVI in High Risk Patients								
	Unadjusted	Adjusted	Unadjusted KCCQ			Adjusted[1] KCCQ Model		
	LS Mean (weighted)	LS Mean (weighted)	Estimate* (95% CI)	T Statistic	P Value	Estimate* (95% CI)	T Statistic	P Value
MCC <4	79.16	74.92						
MCC 4-<6	74.75	72.39	-4.41 (-4.85, -3.98)	-19.80	<.001	-2.54 (-2.97, -2.11)	-11.50	<.001
MCC >=6	68.92	68.91	-10.24 (-10.66, -9.83)	-48.60	<.001	-6.02 (-6.47, -5.56)	-25.70	<.001

*Reference group is MCC < 4.

[1] Adjusted by baseline KCCQ score, age, sex, BSA, lvef, hemoglobin, platelet count, procedure date, eGFRcr, current dialysis, non-white or Hispanic, left main stenosis >=50%, proximal LAD >=70%, Prior MI, endocarditis, prior stroke/tia, carotid stenosis, prior PAD, smoking status, diabetes, NYHA class IV, atrial fib/flutter, conduction defect, severe chronic lung disease, home oxygen use, hostile chest, porcelain aorta, pacemaker, previous ICD,prior PCI, prior CABG, prior cardiac operations, prior aortic valve procedure, prior mitral valve procedure, aortic etiology [degenerative vs. other], valve morphology [tricuspid vs other], moderate/severe aortic stenosis, moderate/severe mitral regurgitation, moderate/severe tricuspid regurgitation, non-femoral access and acuity of TAVR [elective, urgent, shock or inotropes or assist device, emergency or salvage or cardiac arrest].

Supplemental Table 5. Days alive and out of the hospital for MCC-TAVR patients (Full Cohort)							
	MCC Category	Unadjusted			Adjusted[1] Model		
	Median number of days alive and out of hospital	Incidence rate ratio* (95% CI)	Z Statistic	P Value	Incidence rate ratio* (95% CI)	Z Statistic	P Value
MCC < 4	360.0						
MCC 4-<6 vs MCC <4	358.0	0.98 (0.98, 0.98)	-35.1	<0.001	0.99 (0.99, 0.99)	-21.0	<0.001
MCC >=6 vs MCC <4	349.0	0.94 (0.95, 0.94)	-64.7	<0.001	0.96 (0.96, 0.96)	-48.2	<0.001

*Reference group is MCC < 4.

[1] Adjusted by age, sex, BSA, lvef, hemoglobin, platelet count, procedure date, eGFRcr, current dialysis, non-white or Hispanic, left main stenosis >=50%, proximal LAD >=70%, Prior MI, endocarditis, prior stroke/tia, carotid stenosis, prior PAD, smoking status, diabetes, NYHA class IV, atrial fib/flutter, conduction defect, severe chronic lung disease, home oxygen use, hostile chest, porcelain aorta, pacemaker, previous ICD, prior PCI, prior CABG, prior cardiac operations, prior aortic valve procedure, prior mitral valve procedure, aortic etiology [degenerative vs. other], valve morphology [tricuspid vs other], moderate/severe aortic stenosis, moderate/severe mitral regurgitation, moderate/severe tricuspid regurgitation, non-femoral access and acuity of TAVR [elective, urgent, shock or inotropes or assist device, emergency or salvage or cardiac arrest].
